# Identification of Gene Mutations in Atypical Retinopathy of Prematurity Cases

**DOI:** 10.1155/2020/4212158

**Published:** 2020-08-20

**Authors:** Yian Li, Jiakai Li, Xiang Zhang, Jie Peng, Jing Li, Peiquan Zhao

**Affiliations:** ^1^Department of Ophthalmology, Children's Hospital of Fudan University, Shanghai, China; ^2^Department of Ophthalmology, Xinhua Hospital Affiliated to Shanghai Jiao Tong University School of Medicine, Shanghai, China

## Abstract

**Purpose:**

We have observed that some preterm infants whose fundus appears very similar to eyes with familial exudative vitreoretinopathy (FEVR) present with atypical retinopathy of prematurity (ROP). To establish a definitive diagnosis and explore the possible genetic mechanism of atypical ROP, we performed gene sequencing of these cases using next-generation sequencing technology.

**Methods:**

A retrospective review of infants who presented with atypical ROP from October 2013 to February 2017 was performed. The data included gender, gestational age at birth, birth weight, family history, systemic disorders, and age-appropriate ophthalmic examinations. Fundus fluorescein angiography (FFA) of the parents was also performed. Peripheral blood was collected from the patients and their parents to sequence genes. Gene mutations were analysed.

**Results:**

Genetic testing revealed that 9 infants had FEVR-related disease-causing gene mutations. Nine gene mutations were detected; 5 had already been reported, and the other 4 were novel. In the 18 eyes of these 9 patients, 9 eyes exhibited severe ROP. 5 cases had a positive family history.

**Conclusions:**

Gene mutations of low-density-lipoprotein receptor-related protein 5(*LRP5*), frizzled-4(*FZD4*), Norrie disease protein (*NDP*), and tetraspanin-12(*TSPAN12*) may play a role in the pathogenesis of ROP and cause atypical ROP or preterm FEVR. The fundus lesions of ROP patients with disease-causing gene mutations were more serious. ROP cases should be carefully differentiated from preterm FEVR cases.

## 1. Introduction

Retinopathy of prematurity (ROP) is a complex retinal vascular disorder, and its phenotype has many similarities with familial exudative vitreoretinopathy (FEVR). Both diseases are based on abnormal development of retinal blood vessels, so there are secondary retinal complications, including retinal folds and detachment. Retinopathy in premature infants can be regarded as the arrest of normal development of retinal nerve and blood vessels, and its final pathological compensatory mechanism leads to abnormal retinal vascularisation [1]. FEVR is a disorder of retinal blood vessel development principally affecting retinal angiogenesis, leading to incomplete vascularisation of the peripheral retina and poor vascular differentiation [2]. However, emerging genetic research and fluorescein angiographic evidence suggest a margin of overlap between the two [3].

Traditionally, when clinical manifestations are difficult to diagnose, the only strategy to distinguish between diseases has been through clinical history. ROP is a disease that occurs in premature infants and generally has no genetic predisposition. However, FEVR tends to be inherited and can be observed in affected infants at full term. In clinical work, we have found a series of atypical ROP cases appearing very similar to FEVR. To confirm the diagnosis and explore the genetic mechanism of atypical ROP, we used second-generation sequencing technology to carry out gene detection in these cases.

## 2. Materials and Methods

This retrospective study was based on the tenets of the Declaration of Helsinki. Written informed consent was obtained from all patients or their guardians. From October 2013 to February 2017, data were collected for infants who presented with atypical ROP at the Xinhua Hospital Affiliated to Shanghai Jiao Tong University. Next-generation sequencing technology (NGS; MyGenostics, Beijing, China) was used to perform gene sequencing in these cases. We identified a series of ROP patients (*n* = 9) with FEVR-associated mutations.

Data were collected for gender, race, gestational age (GA) at birth, birth weight (BW), dates of fundus fluorescein angiography (FFA), etc. The tests used in the study were performed before treatment.

We followed the methods of our team's FEVR-related topic [4]. All probands were native Chinese individuals. Probands and their relatives underwent comprehensive age-appropriate ophthalmic examinations including intraocular pressure (IOP) measurement, type-B ultrasound, indirect ophthalmoscopy with a 20D noncontact lens (Volk, Mentor, America), and FFA or colour fundus (RetCam 3, America), Heidelberg HRA2 (Heidelberg Company, Heidelberg, Germany), or wide-field fundus (Optomap 200Tx; Optos PLC, Dunfermline, UK) photography. The fundus findings of each proband and his or her relatives were assessed by an experienced retinal disease specialist. Any relative with ROP/FEVR-like fundus was considered to have a positive family history. Disease severity was further assessed and classified in accordance with the ROP staging system described in Hellstrom et al [1].

Peripheral blood samples were collected from all family members who signed the consent from. NGS genetic analysis was carried out on frizzled-4 (*FZD4*), low-density-lipoprotein receptor-related protein 5 (*LRP5*), Norrie disease protein (*NDP*), tetraspanin-12 (*TSPAN12*), and zinc finger protein 408 (*ZNF408*), which are known to be related to FEVR (kinesin family member 11 (*KIF11*) and the *β*-catenin gene (*CTNNB1*) were not screened in this study because of their low incidence in FEVR). The variants previously reported in the Human Gene Mutation Database (HGMD; http://www.biobinternational.com/product/hgmd) were identified as pathogenic. The public databases were used to filter loci with allele frequencies (MAFs) greater than 0.05, and the candidate pathogenic mutations were those that led to a change in the gene structure or an amino acid. The pathogenicity of candidate mutations was evaluated by computer simulation prediction. Then, the criteria of the American College of Medical Genetics and Genomics (ACMG) Standards and Guidelines [5] were used to analyse the pathogenicity. Finally, the candidate pathogenic mutations were analysed. The fundus features of ROP patients with mutations were further studied and compared with those of patients with FEVR. The characteristics of these atypical ROP cases were summarized.

## 3. Results

The demographic data of the 9 patients in this study are summarized in Table 1. Eight patients were male, and 1 was female. GA at birth ranged from 28 weeks to 36 weeks (mean: 33.78 weeks), and BW ranged from 1050 grams to 3500 grams (mean: 2324.44 grams).

All patients had binocular involvement. Among the 18 eyes in the 9 cases, 5 eyes (27.78%) were stage 1, 3 (16.67%) were stage 4a, 4 (22.22%) were stage 4b, and 6 (33.33%) were stage 5. We defined “severe ROP” as any case at stage 3. Severe ROP developed in 13 eyes (72.22%) (Table 2).

**Figure 1:**
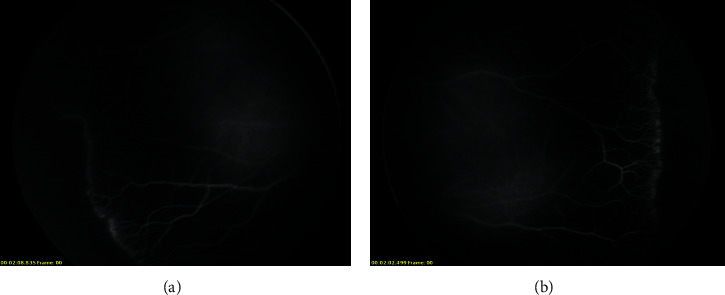
Fluorescein angiography (FA) of case 1. (a) Right eye. (b) Left eye. Both eyes were zone II stage 1 without plus disease. Irregular sprouts of vascularisation at the vascular/avascular junction, distinct pruning of vessels, and pinpoint areas of hyperfluorescence were observed.

**Figure 2:**
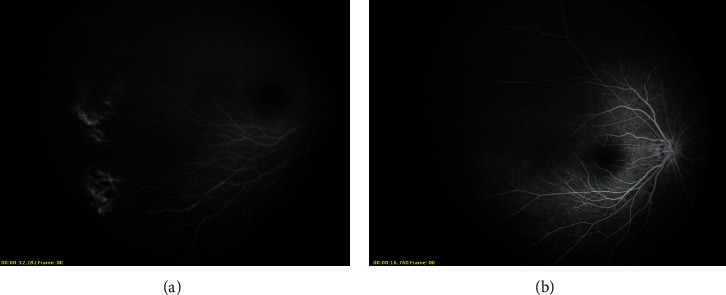
Left eye of case 7. Vascular loops with tangles beyond the edge of vascularisation were observed.

**Figure 3:**
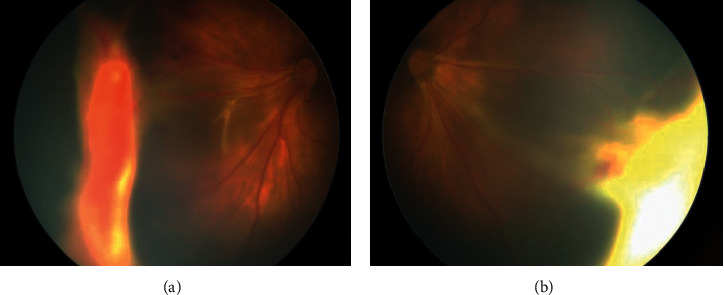
Colour fundus of case 2. (a) Right eye. (b) Left eye. Both eyes were stage 4a accompanied by macular dragging.

**Figure 4:**
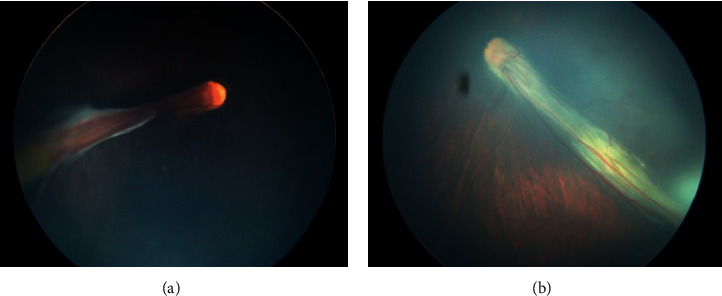
(a) Right eye fundus of case 8. (b) Left eye fundus of case 9. Stage 4b lesions were mostly manifested as falciform fold of the retina.

**Figure 5:**
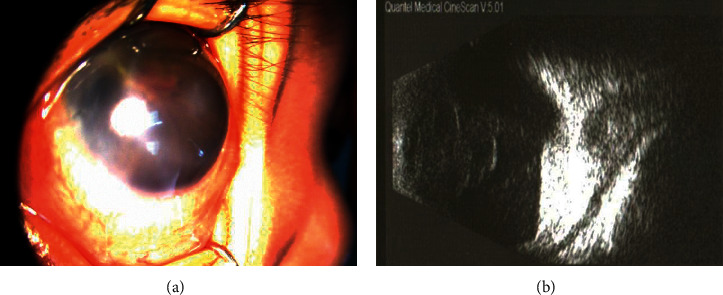
Right eye of case 4. (a) Flat anterior chamber. (b) B-scan of the right eye showed funnel-form retinal detachment.

**Figure 6:**
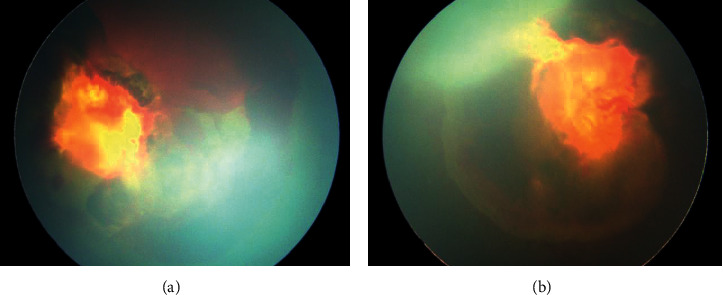
Colour fundus of case 5. (a) Right eye. (b) Left eye. Both eyes showed total retinal detachment with fibrosis and haemorrhage in front of the optic nerve.

Stage 1 lesions were mostly located in zone 2 without plus disease. As observed in the angiogram, irregular sprouts of vascularisation at the vascular/avascular junction, distinct pruning of vessels, and pinpoint areas of hyperfluorescence were observed (Figure 1). In addition, vascular loops with tangles beyond the edge of vascularisation were also noted in one eye (Figure 2). We did not observe typical stage 3 lesions in our cases. Stage 4a lesions were all accompanied by macular dragging (Figure 3). Stage 4b lesions mostly manifested as falciform folds of the retina (Figure 4). Four stage 5 eyes had funnel-form retinal detachment associated with a flat anterior chamber (Figure 5), while the other two stage 5 eyes exhibited total retinal detachment with fibrosis and haemorrhage in front of the optic nerve (Figure 6).Nine variants were found in all the patients (Table 3). We identified 6 known mutations and 3 novel variants [6–10]. Among these, *LRP5* and *NDP* mutations were the most common (3/9, 33.33%), followed by mutations in *FZD4* (2/9, 22.22%) and *TSPAN12* (1/9, 11.11%). All NDP mutations were hemizygous, and the other mutations were heterozygous. At least one computer simulation prediction of each novel variant was “damaging,” “probably damaging,” or “disease-causing,” and the allelic frequency (AF) of these variants was very low (N/A or <0.05) in all databases that we accessed. Therefore, we considered these 3 novel variants (c.2431A > G and c.1434G > A in *LRP5* and c.194C > T in *TSPAN12*) to be likely pathogenic gene mutations. However, obvious ascertainment bias for sporadic cases could not be completely excluded. Further studies to determine the function of these loci are needed to prove this theory.

Of the 9 patients, 5 (55.56%) had a positive family history. *NDP* mutations were paternally inherited. Of the other mutations, four were maternally inherited, and two were paternally inherited.

All patients had no bone, hearing, or other anomalies except for prematurity.

## 4. Discussion

The characteristic of FEVR is failure of peripheral retinal vascularisation during ophthalmic development, which is associated with a spectrum of clinical manifestations. FEVR can be confirmed by genetic testing [11]. To date, five causative genes have been discovered to be related to FEVR, including *LRP5, FZD4, TSPAN12, NDP,* and *ZNF408*. FEVR has Mendelian inheritance [2, 11–14]. ROP is a multifactorial disease that is genetically heterogeneous with multiple alleles with varying magnitudes of effect. The similarity of clinical manifestations between ROP and FEVR further reflects the possibility of the involvement of these genes in ROP pathogenesis.

Recently, several genetic analyses have shown that FEVR-related mutations exist in children with advanced ROP [15–21]. The clinical manifestations of these two diseases are very similar. Both diseases can present with peripheral avascularity, neovascularisation, vitreous haemorrhage, subretinal exudation, vascular dragging, radial retinal folds, and tractional retinal detachments [22–25]. Furthermore, in fundus FA, both FEVR and ROP were thought to appear as abrupt cessation of the capillary network with the formation of scalloped borders and leakage of fluorescein dye from this border [3].

Some scholars believe that most ROP patients display high-fluorescence lesions similar to “popcorn,” abnormal branches (such as circumferential or tangled vessels), local telangiectasis, neovascularisation, and leakage [3]. However, the characteristics of the vascular/avascular junction in FEVR patients include a sudden cessation of capillaries, bulbous vascular endings, venous/venous shunting (rather than arterio/venous), and abnormal increased branches of vessels [26]. In addition, irregular sprouts of vascularisation were observed at the vascular/avascular junction in patients with FEVR in contrast to the vessel profile of classical ROP patients, which was more uniform [3]. Angiography of stage 1 patients in our series showed that they were more likely to have FEVR rather than typical ROP. Only one case (Case 7) showed vascular loops with tangles, which are more inclined to ROP. Nevertheless, ROP is difficult to distinguish from FEVR simply by clinical manifestations.

All nine cases except for two (cases 1 and 2) had a GA of 34 weeks or more and a minimum BW of 2200 grams. China's ROP screening standards are a BW < 2000 grams and a GA < 32 weeks. The GAs and BWs of these seven infants were significantly greater than those of the infants with typical ROP. Furthermore, in four cases (cases 3, 7, 8, and 9), the GAs were 36 weeks, which is close to full term. Vascularisation of the retina begins at approximately 18 weeks of gestation and is complete by 38–40 weeks of gestation [27]. ROP represents the pathological compensatory mechanism of retinal abnormal vascularisation in premature infants due to the arrest of normal development of retinal nerves and vessels. Greater immaturity at birth corresponds to a more aggressive pathological response later. Both a young GA and a low BW are associated with increased severity of ROP [28]. In both animal and human studies, hyperoxia is an important factor that inhibits ROP angiogenesis [29–31]. Studies have shown that among infants with a GA of less than 27 to 29 weeks, ROP was reported in 33% to 73% of infants and severe ROP was reported in 10% to 35% of infants [32–37]. Typical ROP occurs in young-GA and low-BW infants. Of the 14 eyes in the 7 cases, 11 eyes (78.57%) had severe lesions, suggesting that the severity of the lesions may be affected not only by the environment, GA, and BW but also by gene mutations.

Case 1 was born at a GA of 29 weeks with a BW of 1050 g, and both eyes were zone II stage 1 without plus disease. The case was not serious, and according to the characteristics of FFA, we considered this case to be similar to FEVR. Case 2 was born at a GA of 28 weeks with a BW of 1270 g. Both eyes were stage 4a with temporal tractional retinal detachment, proliferation, exudation, and macular dragging. This case is more similar to a typical ROP.

John [3] reported nine cases of premature delivery with features resembling FEVR and introduced a new classification: ROPER (ROP vs. FEVR). However, we believe that these cases cannot be simply classified as one disease. Some cases may be premature FEVR, while others may be ROP combined with FEVR, especially those with more severe disease, which we may call “ROPER.” Family history and genetic testing also support the diagnosis of FEVR. Mutations of FEVR-related pathogenic genes have been investigated in ROP. In one study, mutations in the *FZD4* gene were found in up to 7.5% of patients with severe ROP [38–41]. A study of 421 patients with various vitreoretinopathies found significant correlations between the *FZD4* double missense mutation and both ROP and FEVR [41]. In a study of 53 Japanese ROP patients, 13% of advanced ROP (stage 4 or 5) patients were found to carry mutations in *FZD4* or *LRP5* [40]. According to an American study, 11% of infants with severe ROP had polymorphisms in the *NDP* gene [20]. These studies indicate that the presence of FEVR mutations in ROP may contribute to more advanced retinopathy, which supports our point that ROP with FEVR mutation is more severe, possibly because of ROP combined with FEVR. One hypothesis is that advanced ROP is caused by mutations of genes with less severe functional impact, while FEVR is caused by mutations with greater phenotypic consequences [40]. Recently, an Indian study found a heterozygous variant in the *TSPAN12* gene in a patient with threshold ROP [42]. However, a limitation of these reports (including this study) is that they may have excessively relied on computer simulations to predict the pathogenicity of alleles. Functional analysis is required to determine causation more clearly. In addition, most genetic studies in ROP have been performed on candidate genes. The advantage of candidate gene studies is that in association with a disease, biologic plausibility often exists when selecting certain pathways. The limitation, however, is that only those genes that are believed to be involved are analysed. A genome-wide association study on a larger number of ROP infants is needed.

ROP cases should be carefully differentiated from preterm FEVR cases, especially ROPER and preterm FEVR. However, distinguishing the two diseases only by GA is unreasonable. ROPER presents with indeterminate activation episodes, and FEVR is relatively stable; accurate diagnosis provides guiding significance for treatment. ROPER requires close monitoring for disease progression early and often throughout life, including serial FA and treatment with cryotherapy, laser, and anti–vascular endothelial growth factor (anti-VEGF) injections [3]. However, only patients with FEVR with significant signs of progression or a high risk of progression should accept treatment [43].

## 5. Conclusion

ROP is a complex disease in that evidence suggests that it is influenced by both genetic and environmental factors. Our findings showed that gene mutations of *LRP5, FZD4, NDP*, and *TSPAN12* may play a role in the pathogenesis of ROP and cause atypical ROP or preterm FEVR. The fundus lesions of ROP patients with disease-causing gene mutations are more serious and may be FEVR combined with ROP, and such diseases may be called ROPER. ROP cases should be carefully differentiated from preterm FEVR cases.

## Figures and Tables

**Table 1:** Baseline demographic data.

Patient	Sex	GA at birth (wks)	BW (g)

1	M	29	1050
2	M	28	1270
3	M	36	2950
4	M	35	2500
5	M	34	2250
6	M	34	2200
7	F	36	2500
8	M	36	3500
9	M	36	2700

M, male; F, female; GA, gestational age; BW, birth weight.

**Table 2:** Clinical characteristics of 9 patients and the family members' fundus.

ID	Stage (R/L)	IOP (mmHg R/L)	Family history		
			Father	Mother	Other members

1	1/1	12/10	−	−	N/A
2	4a/4a	6/6	−	−	N/A
3	4a/1	15/15	+	−	N/A
4	5/4b	15/6	−	+	N/A
5	5/5	8/8	−	+	N/A
6	5/5	23/11	−	−	N/A
7	1/5	18/10	−	+	N/A
8	4b/1	9/8	−	−	Brother, −
9	4b/4b	20/13	−	+	N/A

IOP, intraocular pressure; N/A, not applicable; R, right eye; L, left eye; −, normal; +, abnormal fundus.

**Table 3:** Genetic analysis of 9 patients.

Id	Gene	Location	Base changes	Amino acid changes	Gene type	Effect	1000 g	ExAC	Shift	PolyPhen	MutationTaster	ACMG	Source of variation

1	LRP5	exon11	c.2447A > C^*∗*^	p.Q816P	Het	Nonsynonymous	N/A	N/A	D	Ps.D	D.C	Likely pathogenic	Maternal
2	LRP5	exon11	c.2431A > G	p.I811V	Het	Nonsynonymous	N/A	0.00000833	T	P.D	D.C	Uncertain	Maternal
3	LRP5	exon7	c.1434G > A	p.W478X	Het	Stop-gain	N/A	N/A	N/A	N/A	Dc.A	Pathogenic	Paternal
4	NDP	exon3	c.181C > A^*∗*^	p.L61I	Hemi	Nonsynonymous	N/A	N/A	D	P.D	D.C	Pathogenic	Maternal
5	NDP	exon2	c.134T > G^*∗*^	p.V45G	Hemi	Nonsynonymous	N/A	N/A	D	P.D	D.C	Pathogenic	Maternal
6	NDP	exon2	c.134T > A^*∗*^	p.V45E	Hemi	Nonsynonymous	N/A	N/A	D	P.D	Dc.A	Pathogenic	Maternal
7	FZD4	exon2	c.313A > G^*∗*^	p.M105V	Het	Nonsynonymous	N/A	0.0000167	T	Ps.D	Dc.A	Pathogenic	Maternal
8	FZD4	exon1	c.40_49del^*∗*^	p.P14fs	Het	fs	N/A	N/A	N/A	N/A	N/A	Pathogenic	Paternal
9	TPSAN12	exon4	c.194C > T	p.P65L	Het	Nonsynonymous	N/A	N/A	T	B	D.C	Uncertain	Maternal

B, benign; *T*, tolerated; D, damaging; P.D, probably damaging; Ps.D, possibly damaging; D.C, disease causing; Dc.A, disease-causing automatic; A, adenine; C, cytidine; *G*, guanine; *T*, thymine; *Q*, glutamine; P, proline; I, isoleucine; V, valine; W, tryptophan; X, Xaa; L, leucine; E, glutamic acid; M, methionine; fs, frameshift.

## Data Availability

The data used to support the findings of this study have not been made available because of the ethical concerns and patient privacy.
